# A Comprehensive Review of Analysis Strategies for 25-Hydroxyvitamin D_3_: Mechanisms, Platforms, and Future Perspectives

**DOI:** 10.3390/bios16060314

**Published:** 2026-06-01

**Authors:** Dehui Bi, Yiran Cheng, Xinyang Sun, Yuancong Xu

**Affiliations:** Beijing Key Laboratory of Cardiopulmonary-Cerebral Resuscitation Innovation and Translation, College of Chemistry and Life Science, Beijing University of Technology, Beijing 100124, China; bidehui@emails.bjut.edu.cn (D.B.); chengyiran@emails.bjut.edu.cn (Y.C.); sunxinyang@emails.bjut.edu.cn (X.S.)

**Keywords:** 25-hydroxyvitamin D_3_, molecular recognition, biosensor, clinical diagnosis, health assessment

## Abstract

Vitamin D_3_ is an essential fat-soluble vitamin for the human body. Its metabolite, 25-hydroxyvitamin D_3_ (25(OH)D_3_), serves as the primary biomarker to assess vitamin D levels. The monitoring of 25(OH)D_3_ concentration is crucial for human health assessment. While traditional detection methods offer high sensitivity and accuracy, they are operationally complex and costly. This review systematically summarizes the most recent progress in 25(OH)D_3_ detection technologies. Special attention is given to the recognition modes of 25(OH)D_3_ by antibodies, nucleic acids, and molecularly imprinted recognition elements. Subsequently, the design strategies of diverse types of biosensors, including fluorescent, colorimetric, and electrochemical biosensors, are analyzed. Moreover, the development of portable devices, smartphone software, and flexible wearable devices for detection applications is also examined. Biosensing detection platforms are compared from the perspectives of target recognition, signal conversion, signal output, and application scenarios. Additionally, the potential of biosensor detection platforms in clinical diagnosis, health management, and community health surveillance is further investigated. Finally, the future trends of intelligent, portable, accurate, and home-use 25(OH)D_3_ detection systems are delineated. This review offers a comprehensive reference for researchers developing next-generation 25(OH)D_3_ diagnostic sensors and provides insights for the early prevention and treatment of vitamin D deficiency-related diseases.

## 1. Introduction

Vitamin D, an indispensable fat-soluble steroid compound, assumes a pivotal role in upholding calcium and phosphorus homeostasis, regulating bone remodeling, and modulating the functions of diverse organ systems [1,2,3]. Vitamin D manifests in two principal forms, vitamin D_3_ (VD_3_, cholecalciferol) and vitamin D_2_ (VD_2_, ergocalciferol), which jointly constitute the core system for assessing the vitamin D status within the human body. Nevertheless, VD_3_ and its metabolite demonstrate notably higher biological activity compared to VD_2_. Vitamin D insufficiency is widespread across all age cohorts globally and may give rise to diverse health problems, encompassing rickets, elevated infection susceptibility, and cardiovascular disorders, as well as emotional and cognitive impairments [4]. Consequently, in the fields of clinical diagnostics and scientific research, there is an increasing requirement for the quantitative detection and structural analysis of vitamin D and its metabolites. This requirement has propelled the progress of analytical detection technologies, promoting the development of methods featuring higher sensitivity, specificity, and accuracy.

25-Hydroxyvitamin D_3_ (25(OH)D_3_) serves as the primary circulating metabolite of vitamin D (VD) following hepatic hydroxylation. It encompasses both cutaneous synthesis and exogenous intake. Consequently, the concentration of 25(OH)D_3_ mirrors the VD level within the body and can serve as a diagnostic parameter for VD deficiency and a means for therapeutic surveillance [5]. Traditional detection methods for 25(OH)D_3_ include enzyme-linked immunosorbent assay (ELISA), liquid chromatography–mass spectrometry (LC-MS), chemiluminescent immunoassay (CLIA), and high-performance liquid chromatography (HPLC). Although these methods possess distinct advantages respectively, they are still constrained by various issues, including intricate sample preparation, protracted processing durations, elaborate operations, substantial equipment costs, and potential risks related to reagents, which impede their extensive application.

Aptamer, a type of single-stranded oligonucleotide screened from random libraries using the systematic evolution of exponentially enriched ligand (SELEX) technique, specifically recognizes target molecules with high affinity [6,7]. It has been extensively employed as recognition elements within the domain of analysis and detection. Aptasensors are highly sensitive biosensors that can transform into diverse detectable signals (e.g., optical or electrical signals) through the conformational alterations of the aptamer upon binding to the target molecule. In the context of the continuous technological advancement, novel detection methods, such as portable devices, smartphone-integrated platforms, and flexible and wearable devices, are emerging. These technologies not only overcome the spatiotemporal limitations of traditional methods, making detection more convenient and efficient, but also provide strong support for personal health management and public health surveillance.

**Figure 1:**
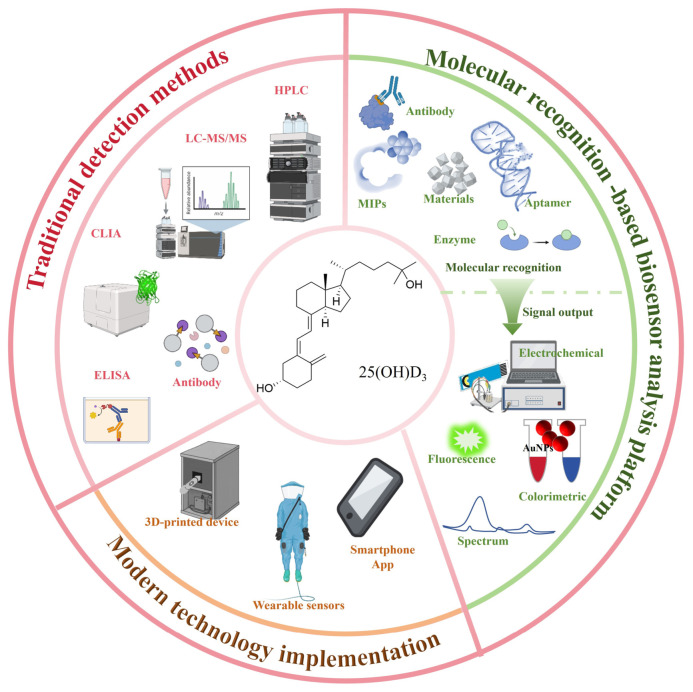
Summary of 25(OH)D_3_ detection methods.

Although the impacts of VD deficiency have been investigated, there lacks a comprehensive review that systematically summarizes the diverse detection platforms targeting 25(OH)D_3_. This review delineates the recognition mechanism, detection performance, and practical applications of each method via different molecular recognition elements, namely antibodies, aptamers, and molecularly imprinted polymers. The limitations of traditional methods are comprehensively examined, and the trends of aptasensor and other emerging detection technologies are explored (Figure 1). This review aims to offer novel insights and directions for the further development of 25(OH)D_3_ detection technologies, thus contributing to the progress of precision medicine and personalized health management.

## 2. Overview of 25(OH)D_3_

### 2.1. Anabolic and Catabolic Pathways of 25(OH)D_3_ in the Body

**Figure 2:**
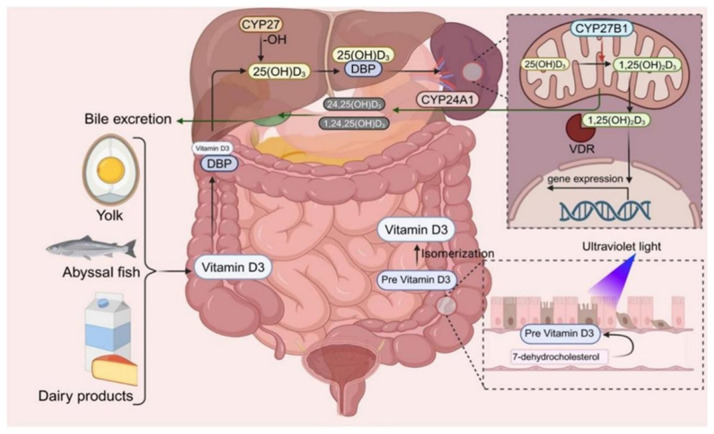
The synthesis and metabolic process of 25(OH)D_3_ in the body. VD_3_ in food is absorbed into the circulatory system through the intestine and converted to 25(OH)D_3_ in the liver.

The human organism obtains VD_3_ from animal-based food sources, including deep-sea fish, egg yolks, and fortified dairy products. These dietary items generally contain abundant 7-dehydrocholesterol, which exists in the basal layer of the epidermis and enters the circulatory system through intestinal absorption. When exposed to ultraviolet (UV) B radiation (290–315 nm), 7-dehydrocholesterol undergoes photolytic decomposition to generate pre-vitamin D_3_, which subsequently undergoes isomerization into VD_3_ (cholecalciferol) under the effect of body temperature [8]. 25(OH)D_3_ is biosynthesized in the liver through the hydroxylation of VD_3_ catalyzed by the enzyme cytochrome P450 27 (CYP27). It functions as the principal circulating form for evaluating vitamin D status within the body [9]. The vitamin D-binding protein (DBP), responsible for transporting VD_3_ to the liver, exerts a crucial function in the circulation of vitamin D. Once 25(OH)D_3_ binds to DBP, it is conveyed to the proximal tubular cells of the kidneys. Within the mitochondria, 25(OH)D_3_ 1α-hydroxylase (CYP27B1) catalyzes the hydroxylation of 25(OH)D_3_ at the 1α position, transforming it into the active form, 1,25-dihydroxyvitamin D_3_ (1,25(OH)_2_D_3_) [10]. 1,25(OH)_2_D_3_ subsequently binds to the vitamin D receptor (VDR) within target cells. Therein, it modulates gene expression to facilitate calcium and phosphorus absorption in the intestine, promote bone mineralization, and regulate immune function. Ultimately, CYP24A1 hydroxylates 25(OH)D_3_ and 1,25(OH)_2_D_3_ to yield their inactive forms, 24,25(OH)_2_D_3_ and 1,24,25(OH)_3_D_3_, which are eventually excreted in the bile [11] (Figure 2).

### 2.2. Diseases Associated with 25(OH)D_3_ Deficiency

25(OH)D_3_ represents the principal metabolite of VD_3_ within the human organism. The concentrations of 25(OH)D_3_ are modulated by parathyroid hormone, blood calcium, and blood phosphorus, and its plasma concentration functions as a crucial indicator for evaluating the VD_3_ status in the body. Based on serum levels, the VD_3_ status can be categorized as deficient (<50 nM), insufficient (50–75 nM), or sufficient (≥75 nM) [12,13,14]. In pediatric populations, a deficiency of 25(OH)D_3_ stands as a predominant etiology of rickets. This insufficiency leads to a reduction in the intestinal absorption of calcium and phosphorus, thereby resulting in diminished blood concentrations of these minerals. Consequently, bone development is impaired, leading to skeletal deformities, including a quadrangular skull, pigeon chest, funnel chest, genu varum, or genu valgum. Additional manifestations may encompass hyperhidrosis, night terrors, and irritability. In adult populations, especially pregnant women, lactating females, and the elderly, a lack of 25(OH)D_3_ impedes the normal mineralization of the bone matrix, leading to bone softening and pain, and thereby heightening the susceptibility to fractures. Patients frequently encounter generalized bone pain and weakness, particularly in the lower back and extremities, significantly impacting their quality of life. Insufficient and deficient levels of 25(OH)D_3_ are strongly correlated with an elevated risk of various diseases, affecting systems including the skeletal, respiratory, reproductive, immune, metabolic, and cardiovascular systems. A deficiency can increase the incidence of these diseases by 30–50% [15,16,17,18,19,20,21].

Recent research has underscored the substantial role of 25(OH)D_3_ in the pathophysiology of coronavirus disease 2019 (COVID-19). A high prevalence of vitamin D deficiency is observed among patients with severe COVID-19, which elevates the risk of intensive-care-unit admission and mortality. Adequate elevation of serum 25(OH)D_3_ levels may enhance the prognosis of patients with COVID-19 [22,23,24]. Consequently, supplementing VD and regularly monitoring its levels represents an economical, accessible, and safe strategy for the prevention and treatment of related diseases, which holds considerable significance [25]. In this context, the development of a highly efficient and precise 25(OH)D_3_ aptamer biosensor is crucial for clinical diagnosis and possesses significant value in formulating early prevention and treatment strategies.

## 3. Traditional Methods for 25(OH)D_3_ Detection

### 3.1. Precision Detection Techniques Based on Instrumental Analysis

High-performance liquid chromatography (HPLC) is highly suitable for the separation and detection of 25(OH)D_3_ in biological samples. When coupled with an ultraviolet–visible (UV-Vis) diode-array detector, accurate detection of 25(OH)D_3_ could be realized. For instance, the limit of detection (LOD) could reach up to 2.92 nM. Consequently, the HPLC-UV method for determining 25(OH)D_3_ levels had been widely acknowledged and utilized internationally [26]. Therefore, the HPLC-UV method for detecting 25(OH)D_3_ levels had gained extensive international recognition and application. To enhance the accuracy of 25(OH)D_3_ detection in serum, an ultra-HPLC (UHPLC) column packed with pentafluorophenylpropyl stationary phase was employed to conduct high-precision analysis of deuterium-labeled 25(OH)D_3_. The limits of quantitation/limits of detection were 4.61/1.38 nmol/L for 25(OH)D_3_ [27]. Mass spectrometry (MS) quantifies the mass-to-charge ratio of charged particles to ascertain crucial information, including the composition, structure, and relative content of a substance. Jiang et al. devised a detection technique for 25(OH)D_3_, which was based on immunoaffinity purification in combination with MS. This method utilized antibody-modified magnetic beads to selectively enrich 25(OH)D_3_ from complex biological samples and precisely identify via MS [28].

Liquid chromatography–tandem mass spectrometry (LC-MS/MS) showcases the complementary merits of chromatography and mass spectrometry, integrating the high separation capacity of chromatography for intricate samples with the high selectivity, sensitivity, and the capacity of MS spectrometry to offer relative molecular-weight and structural information. LC-MS/MS has been extensively utilized in the analysis of 25(OH)D_3_ and is regarded as the optimal approach for determining serum 25(OH)D_3_ levels, as it can separate and precisely quantify both 25(OH)D_2_ and 25(OH)D_3_ [29,30,31,32,33,34,35]. Accurate detection of 25(OH)D_3_ in serum was achieved using 2D-UPLC separation coupled with MS/MS [29]. Wang et al. precipitated proteins in serum with acetonitrile, centrifuged and derivatized with diacetoxyiodobenzene and DMAT, and achieved quantitative detection of 25(OH)D_3_ using LC-MS/MS, which was successfully used to analyze the levels of metabolites in the serum of 109 healthy individuals. The LOD for the method was 12.5 pM [30]. Lin et al. innovatively employed electrospray ionization in positive-ion mode to quantify the serum levels of 25(OH)D_3_ in diabetic patients via an ACE5 C18 column under a methanol gradient, thereby attaining effective separation. The limit of quantification of 25(OH)D_3_ was 1.25 nM in this study. This offers data support for the research on the association between diabetes and vitamin D, facilitating the exploration of the pathological mechanisms and preventive strategies of diabetes [34].

Sample pretreatment is a crucial component of instrumental analysis and accounts for over 50% of the time in the LC-MS/MS analysis process. The pretreatment methods for samples analyzed by LC-MS/MS encompass protein precipitation (PPT), liquid–liquid extraction (LLE), and solid-phase extraction (SPE). By leveraging a stable lithium adduct and a highly sensitive tandem mass spectrometer, sample preparation could be streamlined into a single protein precipitation step, obviating the necessity for derivatization. The achieved limit of quantification was at a level of 37 pM. And this approach could notably enhance detection efficiency and facilitate the quantitative analysis of 1,25(OH)_2_D_3_ in serum samples [36]. SPE was incorporated into the sample processing to enhance the automation level of traditional LC-MS. Deuterated 25(OH)D_3_ was employed as an internal standard. Subsequently, protein precipitation with methanol, centrifugation, and sample purification were carried out. The purification was achieved via a six-step SPE procedure executed by a robotic system. Quantification was then conducted through LC-MS combined with MS/MS in multiple-reaction-monitoring mode. The lower limit of quantitation was 4.0 nM for 25(OH)D_3_. Tailored for routine laboratory applications, this method enables a daily throughput of 300 samples, reduced manual operations to a minimum, and guaranteed efficient and accurate detection [37]. In SPE preprocessing, the packing material played a crucial role in the extraction of target analytes. Precise detection could be accomplished by selecting SPE column packing with suitable adsorption characteristics. Carsten et al. developed an LLE-LC-MS/MS method for the high-throughput detection of 25(OH)D_3_. This was achieved by mixing acetonitrile-denatured serum with a deuterated 25(OH)D_3_ internal standard and performing heptane extraction through a 96-well filtration plate filled with inert diatomaceous earth. The LOD was 10 nM for 25(OH)D_3_ [38]. Zhang et al. first used membrane-emulsified hydrophilic–lipophilic balance (ME HLB) solid-phase extraction microspheres prepared by membrane emulsification technology as SPE packing materials for sample pretreatment, combined with UPLC-MS/MS to achieve accurate detection of 25(OH)D_3_ in serum. The LOD of ME HLB plate for 25(OH)D_3_ was 0.5 nM, meaning that the ME HLB microspheres exhibited better results than other HLBs [39]. Furthermore, Le et al. devised a simplified sample preprocessing approach for the concurrent detection of fat-soluble vitamins (comprising vitamins A, D, and E) through acetonitrile protein precipitation, obviating the necessity for conventional liquid–liquid or SPE treatments. The method demonstrated outstanding linearity (2.5–100 nM), precision, and accuracy when analyzed by LC-MS/MS [40].

In recent years, LC-MS technology has been redefining the scope of analytical science via multi-dimensional innovations. Moreover, its collaborative development with a diverse range of analytical tools offers a more comprehensive solution for the analysis of complex samples [38,41,42,43,44]. LC coupled with ESI or atmospheric-pressure chemical ionization (APCI)–MS/MS demonstrates a high degree of sensitivity, specificity, and versatility. Higashi et al. developed a detection technology for 25(OH)D_3_ in neonatal dried blood spots based on the principle of LC/ESI-MS/MS, using 4-phenyl-1,2,4-triazolin-3,5-dione and an acetylating reagent for two-step derivatization, combined with an Oasis HLB^®^ SPE column and 25(OH)D_3_ as the internal standard. The developed method facilitated the specific quantification of 25(OH)D_3_ in neonatal dried blood spots, with a limit of quantitation of 7.5 nM [41]. Ahmed et al. developed an ultrasensitive UHPLC-ESI-MS/MS technique for 25(OH)D_3_ in patients with COVID-19, using 2-nitrosopyridine as a derivatizing reagent via a controlled microwave-assisted derivatization reaction. The lower limit of quantitation was 0.05 nM in human plasma [43]. Nevertheless, notwithstanding the high precision, this category of methods exhibits certain limitations. These include relatively substantial sample volume requirements, low throughput, and the necessity for specialized technicians, thus restricting, to some extent, its extensive application.

### 3.2. Rapid Immunoassays Based on Antibody Recognition

The introduction of antibodies targeting 25(OH)D_3_ has enabled the development of more straightforward and rapid immunoassays for the detection of 25(OH)D_3_. Immunoassays founded on antibody recognition can be classified into radioimmunoassays (RIAs), enzyme-linked immunosorbent assays (ELISAs), and chemiluminescent immunoassays (CLIAs). RIA was the initial immunoassay approach employed for the estimation of 25(OH)D_3_. Antibodies specific to 25(OH)D_3_ were prepared, facilitating rapid detection via specific antigen–antibody binding. Owing to its minimal sample requirement, this method was appropriate for scenarios where the sample volume was restricted [45]. Additionally, immunodiagnostic systems radioimmunoassay (IDS-RIA) has often been used to measure the levels of 25(OH)D_3_ in plasma. In this method, 125I-labeled 25(OH)D_3_ competed with 25(OH)D_3_ in the plasma sample for antibody binding sites. The content of 25(OH)D_3_ was then assessed based on the radioactivity intensity of 125I [46]. When compared with LC, RIA can achieve comparable results and may potentially have advantages in terms of operational convenience. However, given that RIA can produce hazardous radioactive waste and involves high detection costs, ELISA technology, which is safer and more cost-effective, has gradually become the mainstream detection technology [47].

ELISA can be classified into four prevalent types: direct ELISA, indirect ELISA, sandwich ELISA, and competitive ELISA. Among these, competitive ELISA obviates the need for sample preprocessing, rendering the detection more flexible and stable. A rapid competitive ELISA for the detection of 25(OH)D_3_ was developed. This was achieved by leveraging biotin-labeled 25(OH)D_3_ and competition antibodies specific to 25(OH)D_3_ in the sample, with chloramine T added as a coupling reagent. The detection limit of this approach reached 0.1 μM, substantially enhancing both the efficiency and reproducibility of the assay. The results obtained from this method were compared with those of the radioimmunoassay method, and it was demonstrated that this method met the detection requirements well [48]. ELISA exhibits exceptionally high accuracy, repeatability, and high specificity. Nevertheless, a limitation persists in its development: specifically, the operational procedures are relatively numerous and time-consuming. Consequently, certain assays integrate highly sensitive chemiluminescent detection techniques with ELISA, thereby facilitating the development of CLIA.

CLIA is a simple, sensitive, and cost-efficient approach for high-throughput quantitative analysis of samples. A series of CLIA assays have been developed for the quantitative determination of 25(OH)D_3_ in human serum that exhibit favorable performance and stability [49,50,51]. Moreover, fully automated CLIA systems have been extensively utilized in clinical diagnostics. Certain diagnostic manufacturers launched automated 25(OH)D_3_ immunoassays, such as products from Abbott (Architect), DiaSorin (LIAISON), IDS (ISYS), Roche (E170, monoclonal 25(OH)D_3_ assay), and Siemens (Centaur). These systems facilitate both qualitative and quantitative analyses of 25(OH)D_3_ in samples, substantially improving the efficiency and accuracy of testing. In automated systems, there exist two categories of antibodies. One is a specific antibody that binds to 25(OH)D_3_, and the other is a secondary antibody labeled with a luminescent substance. When excited, the luminescent substance emits a light signal. The instrument then calculates the concentration of 25(OH)D_3_ in the sample according to the intensity of this light signal [52].

In accordance with the CLIA, the electrochemiluminescence (ECL) assay leverages the robust streptavidin–biotin binding interaction to establish complexes among 25(OH)D_3_, antibodies, and a reporter system. Prominent advantages encompass the avoidance of secondary antibodies, thereby curtailing analysis expenses. Wang and colleagues reported a direct competitive ECL assay. Biotin-25(OH)D_3_ engaged in competition with 25(OH)D_3_ in the sample for binding to the ruthenium-labeled antibody. Subsequently, the competitive antigen was captured by streptavidin-coated magnetic microparticles. These magnetic microparticles were magnetically isolated on the electrode, and chemiluminescence was triggered upon the application of voltage [53]. All of these methods were applicable in routine clinical laboratories. Nevertheless, the disparities between their results and those acquired from LC-MS/MS methods call for the resolution of the standardization problem [54,55].

### 3.3. Summary of the Advantages and Disadvantages of Traditional Methods

There exist diverse traditional approaches for the detection of 25(OH)D_3_, with each presenting distinct advantages and disadvantages. Although HPLC exhibits superior linearity, low detection and quantification thresholds, high recovery rates, and reliability, its extensive application is constrained by multiple factors. These encompass the necessity for large sample volumes, low throughput capacity, the capability to process only a small number of samples per analysis, and the requirement for high-level professional expertise and strict operational procedures. LC-MS/MS offers extremely high precision in detecting 25(OH)D_3_ in serum. Despite improvements that have enhanced its automation level, its elevated equipment costs and ion-suppression effects, which disrupt detection results, undermine data accuracy.

In protein binding assays, outcomes from different protein binding techniques may display certain variations. RIA shows advantages under specific criteria; however, in practical applications, the variability among methods complicates clinical diagnoses. ELISA exhibits superior accuracy, repeatability, and specificity and can detect free 25(OH)D_3_. Nevertheless, ELISA harbors a potential risk of cross-reactions, as other substances in the sample may interfere with the detection process. Moreover, the sensitivity of this method may be inadequate in complex samples or when detecting extremely low concentrations, thereby affecting the reliability and accuracy of the results. CLIA is an automated detection method; however, discrepancies exist between its results and those of LC-MS/MS, and the absence of standardized protocols complicates cross-laboratory comparisons.

## 4. Biosensor Analysis Platform for 25(OH)D_3_ Detection

Conventional methods typically exhibit complexity, entailing labor-intensive and time-consuming procedures for sample preparation and storage. In the past few years, biosensor-based detection technologies have emerged as a focal point of research. Owing to their merits of high sensitivity, rapid response, and portability, they present an appealing substitute for traditional chromatography and immunoassays. At present, researchers have successfully devised numerous biosensors for the detection of 25(OH)D_3_, providing novel technological approaches and prospects for relevant detection applications.

Biosensor technology is a technique for detecting biological substances by converting their concentrations into readable signals. Its core principle is founded on the synergistic interplay between biological recognition elements and signal converters. The system acquires information via specific biological reactions and employs physical or chemical transducers to convert biological signals into quantifiable signals, thus facilitating the detection of target substances. Its working principle is generally classified into three principal components: signal recognition, signal conversion, and signal output. In the signal recognition stage, diverse methods are employed to detect 25(OH)D_3_, such as antigen–antibody binding, aptamer binding, molecular imprinting, and other techniques for capturing and recognizing 25(OH)D_3_. Signal conversion is usually integrated with signal output. For instance, identified signals can be converted into optical signals, electrical signals, etc. Future advancements should incorporate more intelligent and convenient approaches for signal output.

### 4.1. Antibody-Based Biosensor for Detecting 25(OH)D_3_

Immunometric assays employing specific antibodies were the primary techniques that guided the design of immunosensors. In these techniques, monoclonal and polyclonal antibodies were generally prepared using a hapten of 25(OH)D_3_, which was conjugated to a carrier protein at the 3-position. Nevertheless, these antibodies exhibited a lack of specificity for the A-ring structure of the metabolites, as evidenced by a high degree of cross-reactivity with 1α,25-dihydroxyvitamin D_3_, whereas the side-chain structure was recognized appropriately [56]. The utilization of hapten carriers conjugated with metabolites containing A rings and side chains can generate highly specific antibodies. Based on this, Kobayashi et al. linked 11α-hemiglutaryloxy-25(OH)D_3_ with bovine serum albumin to form a new immunogenic conjugate, which had induced three polyclonal antibodies that exhibit high affinity for 25(OH)D_3_ (Ka = 0.96–2.6 × 10^9^ M^−1^) [57]. With the progress of technology, antibody preparation had become increasingly sophisticated, and the antibodies employed in detection are predominantly commercial products.

Antibody-based detection of 25(OH)D_3_ predominantly transforms molecular recognition into electrochemical signals via alterations in electron transfer throughout the detection procedure. The electrochemical immunosensor designed for 25(OH)D_3_ is capable of utilizing three signal output techniques: differential pulse voltammetry (DPV), square wave voltammetry (SWV), and electrochemical impedance spectroscopy (EIS).

**Figure 3:**
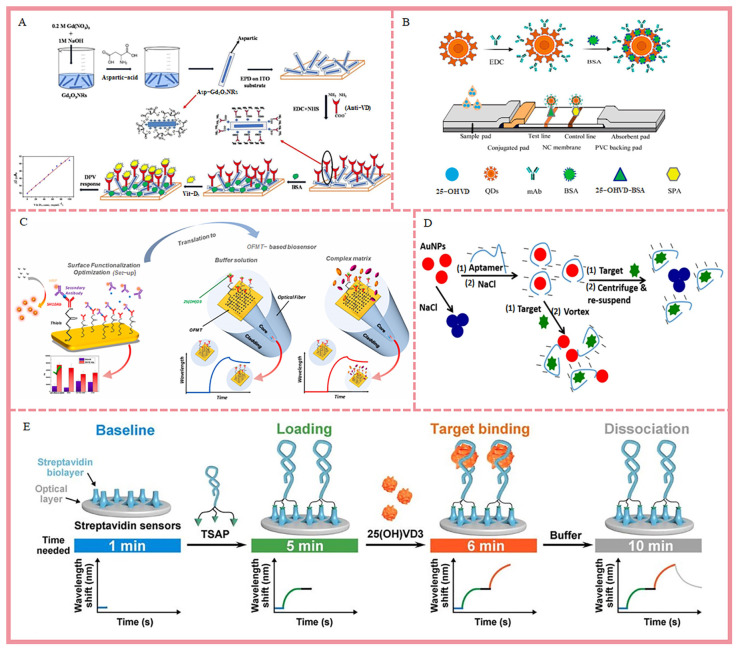
Construction and detection mechanisms of immunosensor and aptasensor for 25(OH)D_3_. (**A**) An efficient electrochemical biosensor based on Gd_2_O_3_NRs (adapted with permission from Ref. [58]). (**B**) The QDs-FICA (adapted with permission from Ref. [65]). (**C**) A novel label-free Lab-on-Fiber biosensing platform (adapted with permission from Ref. [67]). (**D**) Colorimetric aptasensor based on gold nanoparticles (adapted with permission from Ref. [69]). (**E**) The TSA system (adapted with permission from Ref. [70]).

DPV is well-suited for monitoring subtle electrochemical alterations and represents a highly sensitive and remarkably useful technique for the detection of trace analytes. This electrochemical biosensor based on DPV analysis exhibits substantial enhancements compared to other biosensors in terms of sensitivity, specificity, and detection range for 25(OH)D_3_. For example, Chauhan et al. reported an efficient electrochemical biosensor for 25(OH)D_3_ detection using gadolinium oxide nanorods (Gd_2_O_3_NRs) (Figure 3A). Gd_2_O_3_ NRs were functionalized using aspartic acid (Asp), and subsequently deposited onto a glass substrate coated with indium tin oxide (ITO), resulting in the fabrication of the Asp-Gd_2_O_3_ NRs/ITO electrode. The monoclonal antibody of VD (Ab-VD) was immobilized on the surface of the Asp-Gd_2_O_3_NRs/ITO electrode. When 25(OH)D_3_ bound itself to Ab-VD, it facilitated the charge transfer of [Fe (CN)_6_]^3–/4–^, resulting in a change in current value. The results of the response study exhibited a linear range of 25.0–2500 nM for 25(OH)D_3_ detection, and an LOD of 0.25 nM was obtained. This immunosensor showed a satisfactory response to commercially available VD_3_ oral solution [58]. Anusha et al. simplified the electrode design by using graphene nanoribbon (GNR)-modified electrodes to develop an electrochemical biosensor. They prepared an electrochemical probe by conjugating ferrocene-carbaldehyde (Fc-CHO) with antibodies (Ab-25(OH)D_3_), enabling target recognition and signal generation. DPV was used to monitor the electrochemical signal changes in Fc, allowing the detection of 25(OH)D_3_ within a concentration range of 2.5–2500 nM, with a detection limit of 0.25 nM [59]. SWV can effectively improve background issues. By further optimizing the electroactive layer and working conditions, it can further enhance detection sensitivity. In a flexible, label-free electrochemical immunosensor utilizing tree-like gold dendrites (AuDdrites), SWV achieved ultra-sensitive detection of 25(OH)D_3_ within 15 min, with an LOD of 0.07 nM [60]. Although DPV and SWV are more commonly used for quantitative analysis in electrochemical sensors, EIS can detect detailed changes in the charge transfer resistance (Rct) at the electrode interface under smaller-amplitude alternating voltage. And Rct can reflect the electrochemical changes occurring at the surface of the sensing electrode. Anusha et al. developed a novel label-free electrochemical immunosensor using a GCN-β-CD/Au nanocomposite. During detection, an antigen–antibody complex layer forms, impeding electron transfer and increasing the Rct value, thereby enabling accurate measurement of 25(OH)D_3_ in serum samples. The detection range was 0.25–1250 nM, with an LOD of 0.02 nM [61]. In addition, chronopotentiometry techniques can also be used for electrochemical response and can be combined with practical and portable electrochemical devices. In a hydrophilic and insoluble electrospun cellulose acetate fiber-based biosensor, 25(OH)D_3_ was analyzed by chronoamperometry response. A conducting paper substrate (RCP) was prepared by depositing reduced graphene oxide onto ivory paper, and decorated with electrospun cellulose acetate fibers (CAEFs) to form CAEF/RCP electrodes. The electrode was subsequently modified with antibodies specific to 25(OH)D_3_, resulting in a robust interaction between the negatively charged CAEFs and the positively charged amine terminus (NH_2_) of Ab-25(OH)D_3_. All electrochemical studies were conducted in a [Fe(CN)_6_]^3–/4–^ solution. The presence of 25(OH)D_3_ induced a significant rearrangement on the electrode surface due to the antibody–antigen interaction, enhancing charge transfer via [Fe(CN)_6_]^3–/4–^ and resulting in increased current. The fabricated electrochemical immunosensor exhibited a detection range of 25–250 nM and a low detection limit of 25 nM. This paper-based biosensor platform, characterized by cost-effectiveness, environmental friendliness, and disposability, holds promise for pioneering new approaches in biological detection [62].

Furthermore, certain antibody-based optical analysis methods are also employed in the detection of 25(OH)D_3_. Lateral flow strips (LFSs) fulfill the requirement for rapid detection, are straightforward to operate, and represent a mainstream technology in optical biosensors. Wen et al. introduced a method based on colorimetric LFS, which captured 25(OH)D_3_ using antibodies, leading to the aggregation of AuNPs at the test line, thereby enabling visual detection [63]. Anandita et al. synthesized CdS@ZIF-67 nanocomposites using cadmium sulfide (CdS) quantum dots and a zeolitic imidazolate framework (ZIF)-67, which were used as reporter molecules to construct a competitive LFS for the detection of 25(OH)D_3_. The CdS@ZIF-67 nanocomposite was a nanoenzyme with peroxidase-like activity. It was coupled with an antibody, and upon binding to the antigen on the test line, produced a distinct colorimetric response in the presence of 3,3′,5,5′-tetramethylbenzidine (TMB) and H_2_O_2_. The LOD was 67.35 nM, and in spiked serum samples, the LOD was 62.23 nM. The CdS@ZIF67 nanozyme-assisted LFSs hold significant potential for developing highly sensitive and portable biosensing platforms. However, to achieve large-scale applications, safety and environmental friendliness might be considered. More secure nanozyme alternatives should be developed to replace CdS [64].

When nanomaterials on the LFS are substituted with fluorescent materials, the LFS can exhibit enhanced sensitivity. Wang et al. developed a quantum dot nanoparticle-based fluorescent immunochromatographic assay (QDs-FICA), achieving rapid visual and quantitative detection of 25(OH)D_3_, which holds significant potential significance for clinical diagnosis and VD-related diseases (Figure 3B). However, the QDs-FICA exhibited cross-reactivity rates of 26%, 11%, and 25% with 25(OH)D_3_, 1,25(OH)D_3_, and 1,25(OH)D_2_, respectively. To achieve specific detection of 25(OH)D_3_, further improvements were needed in the antibody [65]. Moreover, the clustered regularly interspaced short palindromic repeat (CRISPR) technology had emerged in the domain of fluorescence analysis, particularly the Cas12a system. Cas12a demonstrated endonuclease activity, which allowed it to randomly cleave single-stranded probes labeled with fluorescent and quencher groups, thus leading to fluorescence alterations upon activation. Sun et al. proposed a novel antibody-controlled Cas12a biosensor, which regulated the activity of the Cas12a trans-cleavage enzyme through antibody–small-molecule binding to achieve highly sensitive detection of non-nucleic-acid small molecules. Upon antibody binding, steric hindrance blocked Cas12a’s recognition of PAM, thereby inhibiting enzyme activity. The serum detection system for 25(OH)D_3_ achieved a sensitivity of 650 pM, with detection completed within 30 min [66].

Surface plasmon resonance (SPR) serves as a dependable platform for label-free and real-time biomolecular interaction monitoring in clinical analysis. Its merits encompass real-time analyte surveillance, label-free and parallel analysis, minimal sample preprocessing, quantitative response, high sensitivity, and reproducibility. Cusano et al. reported a highly sensitive biosensing platform based on plasmonic-enhanced MSs integrated onto the tip of a single-mode optical fiber (OF) (Figure 3C). The platform achieved label-free detection of 25(OH)D_3_ through SPR analysis, with a detection limit of 2.10 nM [67]. Esposito et al. developed an OF biosensor for 25(OH)D_3_ detection based on the principle of fiber Bragg grating sensing, using a double-clad fiber (DCF) with a W-type refractive index profile as the substrate. The sensor optimized the DCF structure through chemical etching to enhance sensitivity to the surrounding medium, coated a nanosized GO layer on the surface of a long-period grating transducer, and grafted antibodies as biological recognition elements. Detection was accomplished via the alteration of the refractive index induced by the binding of antibodies to targets, leading to a shift in the grating resonance band. The biosensor achieved a low LOD below 2.5 nM and demonstrated excellent anti-protein interference performance in complex culture media [68].

Nevertheless, the anti-25(OH)D_3_ antibody demonstrates cross-reactivity with 25(OH)D_2_. This disparity in antibody affinity unavoidably gives rise to false-positive outcomes in the detection of 25(OH)D_3_. This represents a limitation of most immunoassay methods for 25(OH)D_3_.

### 4.2. Aptamer-Based Biosensor for Detecting 25(OH)D_3_

Analogous to antibodies, aptamers exhibit high specificity and affinity for their designated target molecules. Aptamers are short, single-stranded oligonucleotides selected from artificial random nucleotide libraries via the SELEX technique. In the detection of 25(OH)D_3_, the aptamer acted as a custom-designed “biological key”, accurately recognizing and binding to the target molecule. In 2012, Bruno et al. employed SELEX technology to successfully acquire 98 candidate aptamer sequences for 25(OH)D_3_. Via sequence alignment, they identified the core stem-loop structure and designed the first aptamer beacon [71]. In 2017, Wen et al. used an exponential enrichment ligand system evolution method based on non-fixed graphene oxide (GO-SELEX) to develop aptamer sequences capable of highly specific and affinity binding to 25(OH)D_3_. Among these nine aptamer sequences, VDBA14 aptamer exhibited high affinity for 25(OH)D_3_, with a Kd value of 11 nM, making it the most widely applied aptamer in aptamer sensor applications [72]. Subsequently, Wen et al. utilized SELEX and AuNP-based analysis to screen and characterize the first aptamer for 25(OH)D_3_ (without disclosing the sequence information) and combined it with an antibody to achieve competitive capture of 25(OH)D_3_ [63].

The aptamer sequences acquired via SELEX technology generally have a length ranging from 50 to 100 nucleotides. Ascertaining the binding region of the aptamer to the target and the target recognition sites could efficiently eliminate redundant sequences, thus enhancing the probability of three-dimensional conformation formation. Gachpazan et al. reasonably truncated VDBA14, resulting in shorter sequences of VDBA14-23, VDBA14-27, and VDBA14-35, thereby significantly enhancing recognition flexibility. Among them, the VDBA14-35 aptamer containing G-quadruplexes exhibited higher sensitivity and specificity compared to the original VDBA14 [73]. However, previous studies conducted structural analyses using CD spectroscopy, which excluded the possibility that VDBA14 forms G-quadruplexes, instead indicating it adopts a typical B-form DNA double helix. Furthermore, after VDBA14 was bound with 25(OH)D_3_, there was no significant structural change in the aptamer, suggesting that 25(OH)D_3_ may bind to a preformed structure [74]. During the molecular docking process of 25(OH)D_3_ with VDBA14, it was found that they mainly bind through groove structures. The two hydroxyl groups of 25(OH)D_3_ act as hydrogen bond donors and form hydrogen bonds with the aptamer. One hydroxyl group formed a hydrogen bond with the oxygen of the phosphodiester bond between DG-20 and DG-19, while the other hydroxyl group formed a hydrogen bond with the carbonyl oxygen of DG-33 [75].

The aptamer-based biosensor for detecting 25(OH)D_3_ is predominantly based on the transformation of molecular recognition events into optical, electrical, or spectral signals via the fluorescence alterations of the probe, the optical characteristics of nanomaterials, and the electrode electrochemical properties. The primary detection strategies are classified into optical biosensors and electrochemical biosensors. Optical biosensors can be further classified into fluorescent, colorimetric, and spectroscopic biosensors according to their signal transduction mechanisms.

#### 4.2.1. Fluorescence Biosensor

Among diverse biosensors, fluorescent sensors present high sensitivity and a broad dynamic range. Fluorescence-based detection can be classified into direct detection and Förster resonance energy transfer (FRET). In direct detection, fluorescence sensing strategies frequently depend on the specific binding of the target molecule to fluorescent probes (e.g., organic dyes and quantum dots), leading to fluorescence quenching or enhancement. For example, PicoGreen (PG) reagent is an asymmetric cyanine dye that does not emit fluorescence in its free state but exhibits high intensity when bound to double-stranded DNA (dsDNA). In a PG-based fluorescence biosensor designed by Qiao et al., the fluorescent signal of PG generated by the dsDNA formed between the aptamer and its complementary strand. Upon the binding of 25(OH)D_3_, the aptamer underwent structural changes, releasing the complementary strand into a single-stranded state, which resulted in a decrease in fluorescence [76]. Badrakhi et al. designed a novel material, cadmium telluride quantum dots modified with thioglycolic acid (CdTe-TGA QDs), which exhibited high intensity effects and was first applied in the fluorescent quantum dot detection of 25(OH)D_3_. Then the CdTe-TGA QD-based biosensor was developed after functionalization with thiol-25(OH)D_3_–aptamers. When the aptamers bound to 25(OH)D_3_, it caused a conformational change in the aptamers and formed non-conductive complexes. Upon re-excitation by photons, the presence of the non-conductive complexes interfered with the electron transfer process, leading to fluorescence quenching [77]. FRET is a non-radiative energy transfer process that occurs when the donor–acceptor pair is in optimal proximity. On this basis, Badrakhi et al. further improved CdTe QDs and replaced TGA with aptamers to construct a novel high-intensity fluorescent probe for ultrasensitive detection of 25(OH)D_3_. This probe was sequentially assembled from CdTe QDs, thiol-25(OH)D_3_ aptamer, and Cy3-labeled nucleic acid. Energy was transferred from the contact between donors (QDs) to the acceptor (Cy3, cyanine 3), resulting in FRET-mediated quenching of QDs’ emission. When 25(OH)D_3_ bound itself to the aptamer, the contact between QDs and Cy3 was disrupted, enhancing the FRET effect, which lead to a decrease in fluorescence intensity. Thanks to this high-sensitivity fluorescent probe, the LOD for 25(OH)D_3_ reaches 8.05 × 10^−9^ M, demonstrating great potential in the development of fluorescent probes for detecting low-concentration analytes [78]. In addition, graphene oxide (GO) is a one-atom-thick sheet of oxidized graphite with excellent solubility, inertness, and specific surface area, and has become a typical fluorescent quencher in the FERT system. In the GO and fluorescent aptamer-based novel biosensor, FAM-labeled aptamers were adsorbed by π–π interaction on the GO sheets, leading to quenching of the fluorescence due to FRET. Target recognition by 25(OH)D_3_ triggered aptamer release from GO and fluorescence recovery. The biosensor achieved a detection limit of 0.37 µM through changes in fluorescence [79]. In the future, enhancing the specificity of aptamers and alleviating fluorescence limitations to improve the sensitivity and practicality of this method will contribute to the development of chip models and their utilization as point-of-care testing (POCT) devices.

#### 4.2.2. Colorimetric Biosensor

Colorimetric approaches are based on the alterations in visible-light absorption induced by nanomaterials (such as gold nanoparticles, AuNPs) or enzyme-catalyzed color reactions, which facilitate intuitive, naked-eye detection. Typically, the negatively charged aptamer sequence undergoes non-specific adsorption on the surface of AuNPs. When the aptamer specifically bound itself to the target, it triggered a conformational change. This conformational change usually diminishes the affinity of the aptamer for the AuNPs’ surface, resulting in its desorption [80,81,82]. For example, upon binding of aptamers to 25(OH)D_3_, the quantity of nucleic acids on the surface of AuNPs diminishes, resulting in aggregation when exposed to salt ions. The aggregation of AuNPs results in a change in optical properties, turning the solution from red to purple-blue (Figure 3D). This biosensor achieved highly sensitive detection of 25(OH)D_3_, with a detection limit of 1 nM [69]. Another frequently employed colorimetric approach leverages the peroxidase-like activity of nanozymes, which catalyzed the oxidation of TMB to generate a stable and specific colorimetric signal. Lee et al. successfully prepared mesoporous platinum nanozymes (MPNs) with a high specific surface area using a surfactant-assisted self-assembly method. The lattice spacing of MPNs was highly aligned with the platinum crystal plane, resulting in significantly enhanced peroxidase-like activity to catalyze the chromogenic reaction of TMB, achieving a catalytic efficiency 3.8 times greater than that of traditional horseradish peroxidase. Therefore, a competitive immunoassay method was developed for the sensitive detection of 25(OH)D_3_, with a linear range of 4.0–250 nM and an LOD as low as 2.5 nM [83]. This method was simple to operate and suitable for on-site detection, although its sensitivity was limited by the aggregation kinetics of the nanomaterials. Zhang et al. designed a novel nanoenzyme, HOFs-g-C_3_N_4_-CeO_2_ (HCC), and established a colorimetric biosensor for the detection of 25(OH)D_3_. The aptamer VDBA14 and HCC-modified complementary single-stranded DNA (ssDNA) were sequentially assembled on magnetic microspheres (MBs). Upon binding of 25(OH)D_3_ to the aptamer, the HCC-modified ssDNA was displaced from the magnetic microspheres. The released HCC then catalyzed the colorimetric reaction of TMB, resulting in an LOD of 18 nM for 25(OH)D_3_ [84]. Nevertheless, to facilitate the clinical application of this biosensor, specific limitations must be overcome. These include eliminating the comparison between substrate color development and standard color charts, and mitigating the influence of ambient light. Moreover, HCC was not merely an exceptional nanozyme with peroxidase-like characteristics but also an outstanding ECL emitter, which had been successfully utilized in ECL biosensors.

#### 4.2.3. Physical Optical Biosensor

Target substances are analyzed through optical principles, including the investigation of the interactions between aptamers and the substances or their specific spectral features. Illustrations of these principles encompass Fourier-transform infrared spectroscopy (FTIR), biolayer interferometry (BLI), localized surface plasmon resonance (LSPR), and surface-enhanced Raman scattering (SERS). In an aptasensor based on a microcantilever, FTIR is employed to verify the binding of aptamers to 25(OH)D_3_. FTIR was used to detect bases in nucleic acids, which had characteristic peaks in the range of 1200–1800 cm^−1^. In this aptasensor, characteristic peaks of the VDBA14 aptamer were found at 1363, 1531, 1550, and 1750 cm^−1^. After adding 25(OH)D_3_, the FTIR spectrum showed a decrease in or disappearance of the characteristic peaks corresponding to the aptamers. This proved that the interaction between the aptamers and the 25(OH)D_3_ caused the aptamers to detach from the microcantilever surface [85]. SPR technology characterizes molecular-binding events by monitoring refractive index changes on a metal surface in real time. LSPR had recently emerged as a highly efficient method for detecting target molecules owing to its label-free assays, and high sensitivity and selectivity. In an LSPR aptasensor, aptamers were immobilized on gold nanorods (AuNRs) that were free of polyethylene glycol. When 25(OH)D_3_ bound itself to these aptamers, the absorbance peak resulting from the LSPR effect shifted toward longer wavelengths, enabling the direct detection of 25(OH)D_3_. The LSPR aptasensor enabled highly sensitive and selective detection of 25(OH)D_3_ across a broad concentration range, with a detection limit of 0.25 nM [86]. BLI is another optical “dip-and-read” biosensor technique that enables real-time analysis of biomolecular interactions. It provides a direct assessment of binding kinetics and offers high-throughput capabilities. Although BLI has slightly reduced reproducibility, it stands out from SPR with higher throughput. Notably, unlike SPR, BLI does not require a microfluidic system for sample delivery to the biosensor surface, thus minimizing instrument maintenance [87]. In a trident scaffold-assisted aptamer biosensor (TSA) system, the aptamers were immobilized on an optical layer coated with streptavidin (Figure 3E). BLI was employed to analyze the interaction between 25(OH)D_3_ and the aptamer, assessing their binding affinity. In the presence of 25(OH)D_3_, the aptamer region of the TSA system recognized and bound to 25(OH)D_3_ in solution, resulting in a shift in the wavelength of light interference. The TSA system could detect 25(OH)D_3_ over a wide concentration range (17.4–12,800 nM), with a detection limit of 17.4 nM. Furthermore, the TSA system also could monitor 25(OH)D_3_ levels dynamically in living cells, providing an efficient platform for researching drug interactions [70]. LSPR is a nanophotonic phenomenon where metallic nanoparticles or nanostructures interact with incident light. In contrast, SERS is a powerful analytical technique that amplifies molecular Raman signals through proximity to specialized nanostructures [88]. Generally, SERS typically employs noble metals as substrates to amplify the Raman signal through the generation of localized electromagnetic fields in hotspots. Biomimetic materials, which feature high-density hotspots, serve as more efficient SERS substrates. A bio-inspired Ag nanovilli-based sandwich-type SERS aptasensor was developed for the detection of 25(OH)D_3_, leveraging the villi structure. The densely packed Ag nanovilli (AgNV) structure enhances the Raman signal by creating hotspots due to its extensive surface area. A 4-phenyl-1,2,4-triazoline-3,5-dione (PTAD)–methylene blue (MB) complex served as the Raman indicator. In this SERS aptasensor, aptamers and antibodies were used together to create an aptamer/25(OH)D_3_/antibody–PTAD-MB complex on the AgNVs. This facilitated the conversion of vitamin signals into Raman signals. The LOD of the aptasensor for 25(OH)D_3_ was 2.5 pM [89].

#### 4.2.4. Electrochemical Biosensor

**Figure 4:**
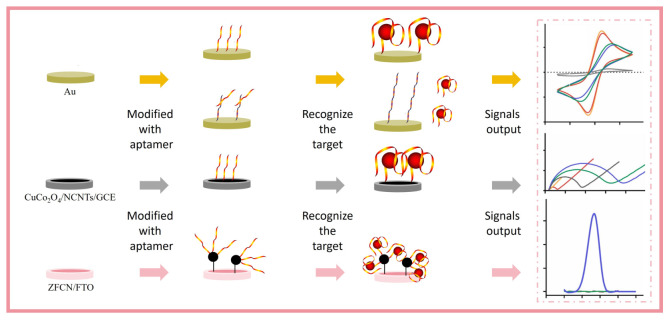
Construction and detection mechanisms of electrochemical aptasensor for 25(OH)D_3_.

Electrochemical biosensors are employed to detect alterations in electron transfer at the electrode interface. These sensors have various merits, including high sensitivity, facile miniaturization, and low cost. Beyond antibodies, aptamers have emerged as an optimal recognition element for electrochemical sensors owing to their high affinity and design flexibility. For instance, Yin et al. developed an electrochemical aptasensor for the detection of 25(OH)D_3_ by immobilizing the VDBA14 aptamer on a gold electrode surface via disulfide bond conjugation (Figure 4). The formation of aptamer–25(OH)D_3_ complexes effectively reduced the electrocatalytic effect of thiolate aptamer on the redox activity of ferri/ferrocyanide. The biosensor monitored electrochemical responses using CV, SWV, and EIS, featuring a linear detection range of 1–1000 nM for 25(OH)D_3_, with an LOD as low as 0.085 nM [90]. In addition to gold electrodes, carbon-based materials [73], AuNP-modified glassy carbon electrodes (GCEs) [75], and nanomaterials [91,92] have been extensively used in electrochemical biosensors, enhancing the electrical conductivity and specific surface area of the electrodes. Meysam et al. developed a novel biosensor based on a CuCo_2_O_4_/nitrogen-doped carbon nanotube (N-CNT) nanocomposite (Figure 4). The aptamer was non-covalently attached to N-CNT via π-π interactions between the heterocyclic rings of nucleotides and the honeycomb structure of the carbon nanotube. When 25(OH)D_3_ bound to the aptamer, it triggered a conformational change, and the concentration of 25(OH)D_3_ was quantified by detecting the attenuation of the current signal of the redox probe [Fe(CN)_6_]^3–/4–^ via DPV. With a sensitivity of 0.063 pM, this biosensor offered high selectivity and affordability, providing an efficient tool for clinical VD detection [73]. To further augment the sensitivity of the aptasensor, a series of signal-amplification strategies, especially nucleic acid amplification strategies, were employed during its construction. Building on previous research, Yin et al. developed a novel electrochemical aptamer biosensor for detecting 25(OH)D_3_ based on catalytic hairpin assembly (CHA) and DNA tetrahedron structures (Figure 4). Hairpin probes were modified on the gold electrode surface using DNA tetrahedrons. In the presence of 25(OH)D_3_, the aptamer beacon recognized the target and formed a complex, exposing a free tail. This tail then participated in a CHA reaction with the Fc-labeled hairpin and the hairpin probe on the gold electrode. Consequently, Fc docked at the electrode surface, facilitating efficient electron transfer and amplifying the electrochemical signal. CV, SWV, and EIS were employed to investigate the electrochemical properties of the developed biosensor. The electrochemical aptasensor demonstrated a wide linear range of 0.1–1000 nM, with an LOD as low as 0.026 nM. It exhibited high selectivity, stability, and reproducibility [93]. Although the label-free strategy simplifies the preparation process, the long-term stability, anti-interference ability in complex biological matrices, and large-scale production costs still require systematic evaluation. In addition to nucleic acid amplification, signal amplification can also be achieved through the modification of nanomaterials. Amandeep et al. developed an ultrasensitive electrochemical aptasensor powered by carboxylate graphitic carbon nitride/zinc ferrite composite (ZFCN) as a support material and palladium-doped cobalt nanoparticles (Pd-Co NPs) as a signal enhancer (Figure 4). Using EIS, the biosensor demonstrated exceptional performance, achieving a detection limit of 0.16 pM and a wide linear range of 2.5–250 nM while remaining stable for up to 60 days [94]. Zhang et al. developed an HCC composite material, modified with aptamers and Au NRs-modified complementary aptamer strands (csDNA), to detect 25(OH)D_3_ via ECL. After 25(OH)D_3_ bound to the aptamer, csDNA was released, blocking the ELC-resonance energy transfer (ECL-RET) between HCC and Au NRs, thereby restoring the ECL response. This method exhibited good response for 25(OH)D_3_ in the linear range of 50–200 nM, with an LOD of 40 nM. In this biosensor, the ECL intensity of the HCC composite was enhanced by more than twice, significantly improving the detection sensitivity. However, the actual energy transfer efficiency between HCC and Au NRs was still limited by the distance, orientation, and surface modification of the nanoparticles, thus requiring further optimization [84].

### 4.3. Detection of 25(OH)D_3_ Based on MIP-Based Molecular Recognition

The application of antibodies is restricted by different factors, including a short shelf-life, high cost, and complex storage conditions. Aptamers also encounter problems such as intricate conformational change design and inadequate selectivity and specificity. Nevertheless, these issues can be surmounted through the utilization of molecularly imprinted polymers (MIPs), which exhibit greater stability and durability. MIPs are polymers specifically engineered to conform to the shape of template molecules. They can be based on host–guest polymerization with target molecules through covalent or non-covalent bonding [95]. Kia et al. developed a molecularly imprinted polymer for VD_3_ using an inorganic–organic adsorbent for the first time. Due to the high structural similarity between 25(OH)D_3_ and VD_3_, the synthesized polymer also exhibits a binding affinity for 25(OH)D_3_. However, VD_3_ was unsuitable as a template for common organic MIP due to its large molecule and two active double bonds. Consequently, the sol–gel method was selected for MIP-based solid-phase extraction of VD_3_. First, the template was captured through an acid-catalyzed reaction within the SiO_2_ network. The organic polymer then underwent polymerization around the network and the template. Finally, after the template was removed, binding sites are formed. During the recognition process, one of VD_3_’s hydroxyl groups became bound to the MIP via hydrogen bonding [96]. However, when MIPs based on hydrogen bonding were used to recognize 25(OH)D_3_, the limited number of functional groups in its chemical structure restricted the number of 25(OH)D_3_ molecules that could bind within the MIP cavity, thus limiting detection sensitivity. To address this issue, researchers often used Diels–Alder reactions to derivatize 25(OH)D_3_, thereby increasing its size and enhancing its binding affinity with the MIP. For instance, Jamilan et al. utilized the reaction between 25(OH)D_3_ and 2-nitropyridine derivatives to generate primary and secondary derivatives of the enantiomers of 25(OH)D_3_, successfully preparing MIPs (styrene-co-N-(3-aminopropyl) methacrylamide). This process prevented interfering compounds from binding to the MIPs’ cavity through hydrogen bonding, significantly enhancing detection specificity [97].

**Figure 5:**
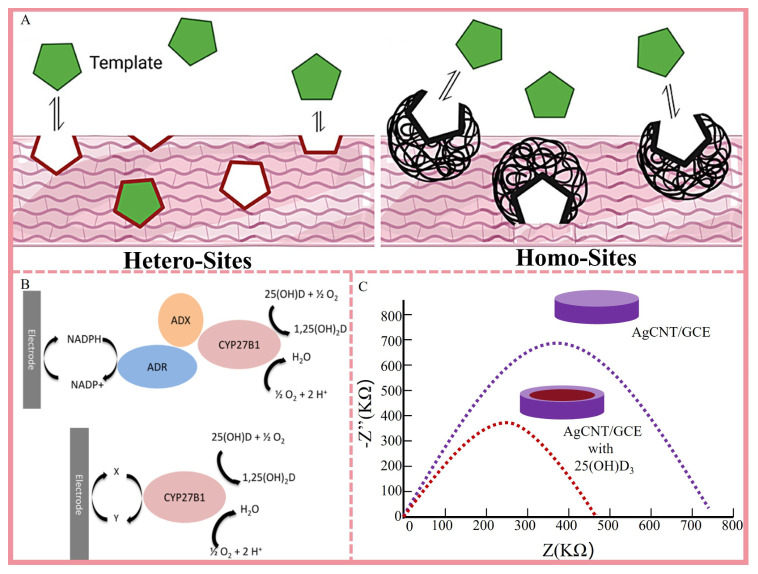
Recognition mechanisms of other aptasensor for 25(OH)D_3_. (**A**) Scheme depicting the heterogeneity of binding sites formed by means of the molecular imprinting process (adapted with permission from Ref. [98]). (**B**) Electron transfer mechanisms in CYP450s (adapted with permission from Ref. [100]). (**C**) Changes before and after the combination of AgCNT/GCE with 25(OH)D_3_.

MIPs are frequently employed as recognition elements in the design of electrochemical biosensors. Leveraging biomimetic principles, they can specifically recognize and sensitively detect target substances. Nevertheless, the detection performance of electrochemical sensors is affected by certain drawbacks of MIPs, such as a limited number of imprinted sites, a sluggish binding rate, and an uneven distribution of binding sites. To enhance the application of electrochemical sensors, MIPs are typically encapsulated on nanoparticles owing to their high conductivity and large surface area (Figure 5A) [98]. Sheikh et al. prepared an MIP layer for 25(OH)D_3_ using a highly conductive polymer, polypyrrole (PPy). They then designed an electrochemical biosensor based on modifying GCE with a nanocomposite of CuCo_2_O_4_/nitrogen-doped carbon nanotubes and phosphorus-doped graphene oxide (CuCo_2_O_4_/N-CNTs/P-GO), which was coated with the 25(OH)D_3_-PPy layer. In the absence of 25(OH)D_3_, the ferricyanide redox reaction proceeded normally on the electrode surface. However, when 25(OH)D_3_ bound to the PPy layer, it obstructed the ferricyanide redox reaction, resulting in a change in the electrical signal. The biosensor demonstrates a linear detection range for 25(OH)D_3_ from 0.002 to 10 μM, with an LOD of 0.38 nM [99].

### 4.4. Detection of 25(OH)D_3_ Based on Enzyme-Based Molecular Recognition

Cytochrome P450 (CYP450) belongs to a class of heme-thiolate monooxygenases in the heme protein superfamily. It possesses the capability to metabolize a wide range of chemicals through various biotransformation reactions and has applications in fields such as biochips, bioreactors, and biosensors. CYP450s are in the ferric (Fe^3+^) form at rest. One-electron reduction of the ferric form leads to the ferrous (Fe^2+^) state, which is widely used in electrochemical design [101]. Design mechanisms for biosensors using enzyme-catalyzed CYP450 reactions include two approaches. One approach involved electron transfer via NADPH–adrenodoxin reductase and adrenodoxin, which allowed the electrochemistry of the NADPH/NADP+ redox couple to be detected at the electrode. The other approach used non-native redox mediators to facilitate direct electron transfer while the enzymes were modified on the electrode. CYP27B1 was a CYP450 enzyme located in the mitochondria that catalyzes the conversion of 25(OH)D_3_ to 1,25(OH)_2_D_3_ in the kidney [10]. However, the level of extracellular expression of CYP27B1 was extremely low and unstable. Ozbakir et al. modified human CYP27B1 by deleting the first 31 amino acids and replacing Ser32 with Met32. Mature recombinant CYP27B1 was successfully expressed in *E. coli* and efficiently converts 25(OH)D_3_ into both 1,25(OH)_2_D_3_ and an isomer of (OH)_2_D_3_ (Figure 5B). The recombinant CYP27B1 was then immobilized on a GCE using pH-regulated Nafion^®^ and tris(1,10-phenanthroline) cobalt (III) chloride (Co(sep)^3+^) as the redox mediator. CV and SWV were then used to characterize the electrode performance by converting the reaction into a detectable electrical signal. This enabled the detection of 25(OH)D_3_ within the physiological concentration range of 12.5–500 nM [100].

From another perspective, 25(OH)D_3_ was converted into 1,25(OH)_2_D_3_ by CYP27B1. 1,25(OH)_2_D_3_ was a high-affinity agonist of the VDR. Therefore, the level of 25(OH)D_3_ could be indirectly determined by observing the interaction between 1,25(OH)_2_D_3_ and VDR. Mano et al. achieved the successful expression of a chimeric protein composed of luciferase and the VDR ligand-binding domain (LBD), and constructed a split-luciferase vitamin D biosensor (SLDB) based on LucC-LBD-LucN. When an agonist was bound to the SLDB, it induced a conformational change in helix 12 (H12) of the LBD. This disruption led to the interruption of the interaction between the LucN and LucC fragments of the split luciferase. Consequently, there was a rapid and considerable reduction in the luminescence intensity of the luciferase. Conversely, upon binding with an antagonist, the light intensity of the luciferase increased instantaneously and significantly. Both 25(OH)D_3_ and 1,25(OH)_2_D_3_ acted as antagonists of VDR. Consequently, while SLDB detected 25(OH)D_3_ in serum, it could not differentiate it from 1,25(OH)_2_D_3_ [102]. To distinguish between the two, Mano et al. enhanced the SLDB design by inserting several LXXLL motif-containing peptides between LucN and LBD to create a new LucN-LXXLL-LBD-LucC biosensor. When 25(OH)D_3_ bound to the LBD, the biosensor emitted a low intensity. However, upon conversion of 25(OH)D_3_ to 1,25(OH)_2_D_3_ by CYP27B1, the biosensor emitted a strong light. This biosensor could respond to 1,25(OH)_2_D_3_ at picomolar levels with increased light intensity, overcoming the sensitivity limitations of previous SLDBs [103].

### 4.5. Detection of 25(OH)D_3_ Based on Nanomaterials

Beyond the conventional recognition methods, label-free (antibody/aptamer/MIPs/enzymes) techniques are also employed in the fabrication of biosensors for the direct detection of 25(OH)D_3_. In this context, the output signal of the biosensor undergoes variation owing to the affinity between specific nanomaterials and 25(OH)D_3_. Fathima et al. developed an Ag-Ag_2_O/CNT-modified glassy carbon electrode (AgCNT/GCE) biosensor for the nanomolar detection of 25(OH)D_3_. The high affinity of AgCNT toward 25(OH)D_3_ led to complex formation and a decrease in the AgCNT/GCE electrode Rct value (Figure 5C). The proposed biosensor exhibited excellent sensitivity in the range of 20–100 nM, with a remarkably low detection limit of 7.9 nM. It demonstrated excellent response stability, repeatability, and reproducibility, with minimal interference, providing a new approach to the label-free detection of 25(OH)D_3_ [104]. Almalki et al. used 25(OH)D_3_–tetraphenylborate ion association complexes to modify graphene quantum dots (GQDs), preparing functionalized GQDs that could be used as fluorescent probes. These probes emitted a high intensity when excited at 350 nm. When the functionalized GQDs interacted with 25(OH)D_3_, the fluorescence was significantly reduced. This alteration in fluorescence facilitated the detection of 25(OH)D_3_ in serum specimens and had been utilized to compare the plasma 25(OH)D_3_ levels between autistic and healthy children [105].

### 4.6. Summary of Biosensor Detection Technologies

Biosensor detection technologies have made significant advancements in the detection of 25(OH)D_3_, demonstrating substantial advantages over traditional methods (Table 1, Table 2 and Table 3). Among these technologies, aptasensors based on optical and electrochemical principles exhibit unique capabilities. In optical aptasensors, fluorescence sensing demonstrated high sensitivity; however, certain mechanisms and applications required further optimization. The colorimetric method was simple but vulnerable to the influence of nanomaterials. Meanwhile, despite its advantages, SPR technology faced challenges in the detection of low-molecular-weight substances. Electrochemical aptasensors exhibit high sensitivity, are easily miniaturized, and have low costs. They can be categorized into labeled and label-free types, each with its own unique advantages. Moreover, biosensors utilizing antibody and MIPs recognition also display distinct characteristics. These diverse biosensor types, whether aptamer-based or operating under other recognition mechanisms, together form a comprehensive system for the detection of 25(OH)D_3_. Their continuous research and application constantly expand the scope of 25(OH)D_3_ detection, offering broader prospects for clinical diagnosis, health monitoring, and other fields. The advent of biosensors has provided a more convenient and rapid approach for detecting 25(OH)D_3_. Nevertheless, most existing detection techniques remain confined to the laboratory phase. With the increasing daily demand and technological advancement, the limitations of traditional biosensor detection methods have become more prominent, while the advantages of emerging detection technologies are attracting increasing attention.

**Table 1:** Performance comparison of different biosensors.

Biosensor	Recognize	Linear Range	LOD	Reference
Electrochemical immunosensor	Antibody on the Asp-Gd_2_O_3_NRs/ITO electrode	25.0–2500 nM	0.25 nM	[58]
Electrochemical immunosensor	Antibody on the GCN-β-CD/Au nanocomposite	0.25–1250 nM	0.02 nM	[61]
Optical immunosensor	Antibody on the CdS@ZIF-67 nanocomposite	/	67.35 nM	[64]
OF biosensor	Antibody and double-clad fiber	2.5–2500 nM	2.5 nM	[68]
Fluorescence aptasensor	Aptamer and CdTe QDs	0.06–6 × 10^7^ nM	8.05 nM	[78]
Colorimetric aptasensor	Aptamer and mesoporous platinum nanozymes	4.0–250 nM	2.5 nM	[83]
LSPR aptasensor	Aptamer and gold nanorods	0.25–2.5 × 10^5^ nM	0.25 nM	[86]
SERS aptasensor	Aptamers, antibodies, and Ag nanovilli	0.0025–250 nM	2.5 pM	[89]
Electrochemical aptasensor	Aptamer on a gold electrode	1–1000 nM	0.085 nM	[90]
Electrochemical aptasensor	Aptamer with CHA on a gold electrode	0.1–1000 nM	0.026 nM	[93]
Electrochemical biosensors	MIPs and CuCo_2_O_4_/N-CNTs/P-GO	0.002 to 10 μM	0.38 nM	[99]
Electrochemical biosensors	CYP27B1 enzyme on a GCE	12.5–500 nM	/	[100]
Electrochemical biosensors	AgCNT and GCE	20–100 nM	7.9 nM	[104]

**Table 2:** Biosensor vs. chromatography.

Comparison Dimensions	HPLC/LC-MS	Biosensor	Advantages of Biosensor
Sensitivity [30,36,106,107]	High—at the pM level	Moderate to high—it can reach the fM level after optimization	Signal enhancement by nanomaterials—such as gold nanoparticles and quantum dots
Detection speed [106]	Slow—30 to 60 min for a single sample	Fast—5 to 15 min	Real-time monitoring capability, which is suitable for dynamic analysis
Equipment cost [106]	Costly—the cost of the instrument plus maintenance exceeds 100,000 US dollars	Low—the cost of the miniaturized biosensor is less than 5000 US dollars	Suitable for primary healthcare and home-based testing
Sample pretreatment [106]	Complex—extraction and derivatization are required	Simple—it can directly detect saliva and whole blood	Reduces human errors and improves throughput
Portability	None—fixed laboratory equipment	High—handheld or wearable devices	On-site detection—for example, outdoor health screening

**Table 3:** Biosensor vs. traditional immunoassay methods.

Comparison Dimensions	CLIA/ELISA	Biosensor	Advantages of Biosensor
Recognition element [108]	Antibody—prone to inactivation, large batch differences	Diversified—aptamers, enzymes, molecularly imprinted polymers	Resistant to high temperature, acid and alkali, long-shelf-life aptamers > 1 year
Detection window period [109]	Reaction needs to be terminated (endpoint method)	Real-time continuous monitoring—for example, surface plasmon resonance technology	Dynamically track metabolic changes (e.g., postoperative recovery of vitamin D)
Multiplex detection [110]	Limited-dependent on a combination of multiple antibodies	Easy to achieve—multichannel biosensor array	Simultaneously detect related indicators such as 25(OH)D_3_, calcium, PTH, etc.
Regenerative capacity [111]	Disposable	Reversible binding—aptamer biosensor can be reused > 50 times	Reduce the cost of a single test < 1 dollar per test
Anti-interference ability	Prone to interference from rheumatoid factors	Anti-pollution interfaces can be designed—for example, anti-protein adsorption coatings	Directly detect complex samples—for example, undiluted serum

## 5. Portable and Intelligent Equipment-Assisted Biosensor for 25(OH)D_3_ Detection

Laboratory-based high-precision detection technologies are increasingly unable to fully meet the demands for convenient, rapid, and real-time testing. POCT, which can promptly generate test results on-site, is characterized by simple operation and portable equipment. It notably improves the efficiency and timeliness of testing, thus emerging as a vital development direction for modern detection technology. In the field of 25(OH)D_3_ detection, the integration of intelligent and portable devices has instigated a revolutionary change in testing methods. These novel detection technologies not only overcome the limitations of time and space but also make the detection process more streamlined and effective, providing significant support for personal health management and public health surveillance.

### 5.1. Portable Device Detection Platform

**Figure 6:**
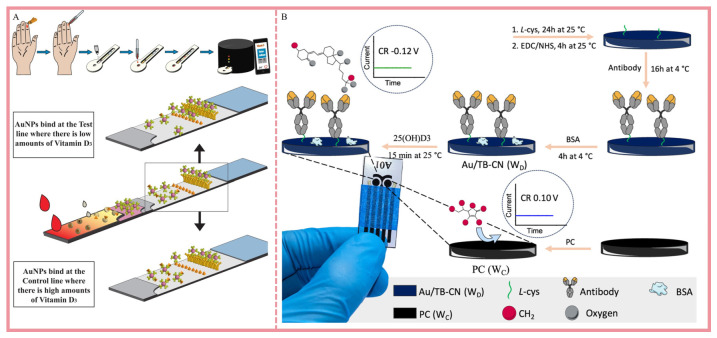
Construction and detection mechanisms of portable and wearable devices for 25(OH)D_3_. (**A**) A portable and smartphone platform for the visual detection (adapted with permission from Ref. [112]). (**B**) A screen-printed chip for wearable sensors (adapted with permission from Ref. [115]).

With the progress in POCT research, paper-based biosensors, particularly LFS, have emerged as rapid detection instruments. They present multiple benefits for users, such as cost-efficiency, flexibility, user-friendliness, and portability. Vemulapati et al. devised a diagnostic testing system for the precise and quantitative evaluation of 25(OH)D_3_ using a smartphone-assisted portable imaging device. This diagnostic testing system comprised a competitive type LFS, a custom-fabricated cassette for housing the test, and a portable reader (TIDBIT, Figure 6A). In this LFS, when 25(OH)D_3_ was present, AuNPs were captured by the test line; conversely, they were captured by the control line, as the control line was pre-deposited with 25(OH)D_3_. The TIDBIT encompassed a complementary metal–oxide–semiconductor camera, a focusing lens, light-emitting diodes (LEDs), a Raspberry Pi board, a rechargeable lithium-ion battery, a slide-out cassette tray, and a three-dimensional (3D)-printed light-impenetrable container. Owing to the 3D-printed technology, compact smartphone imaging devices had been devised, which effectively integrated the advanced biosensor technology with the convenience of contemporary mobile applications [112]. Weng et al. devised a portable paper-based microfluidic device for the expeditious quantification of 25(OH)D_3_. The device incorporated a smartphone accessory, a specialized application, and LFS. The accessory was equipped with an amber LED (λ = 592 nm) to illuminate the LFS during the analytical process. To more effectively actualize the practicality and precision of the portable device, the design emphasis was placed on the hardware within the device. This hardware comprised an integrated sensor network consisting of parameter monitoring apparatuses, environmental monitoring sensors, gateways, local storage units, and a user interface. This portable device accomplished the quantitative analysis of 25(OH)D_3_ by assessing the luminance disparity between the detection area and the reference area on the LFS [63].

Electrochemical biosensors exhibit considerable potential in terms of high sensitivity, rapid response, stability, and instrument miniaturization. Through electrode improvement, they can be developed into portable devices. For instance, flexible electrodes can be employed in wearable devices. Moreover, the integration of microfluidic technology with electrochemical biosensors has facilitated device fabrication, which can effectively reduce costs, minimize reagent consumption, and better fulfill the requirements of portable device detection. Chauhan et al. developed a novel microfluidic nanobioplatform (MNBP) by depositing ITO on a glass substrate and incorporating gadolinium nanoparticles (GdNPs) to enable the precise detection of 25(OH)D_3_. Photoresist materials were applied to the surfaces of Si-wafers and ITO substrates. Leveraging CleWin software, master molds with microfluidic channel patterns and W-R-C three-electrode system patterns were then fabricated. Polydimethylsiloxane (PDMS) was cast onto the Si-wafer mold to generate a PDMS substrate equipped with 200 μm microfluidic channels. The ITO mold was etched using an ITO etchant to create the three-electrode system. GdNPs were deposited in the W region to prepare CS@Gd NPs/ITO, and subsequently, its surface was functionalized with antibodies. After blocking with BSA, this was fabricated into a working electrode, BSA/AB/CS@Gd NPs/ITO. Finally, PDMS was combined with ITO to form the MNBP through semi-covalent bonds via UV/ozone plasma treatment. This portable MNBP facilitated the POCT of 25(OH)D_3_ within the physiological range, exhibiting an LOD of 11 nM and an analysis time of 30 min, rendering it highly appropriate for rural and resource-constrained areas [113].

Furthermore, SERS is an effective tool for developing POCT, particularly when integrated with portable devices. It offers significant advantages, such as reduced sample pretreatment needs, higher diagnostic accuracy, and lower costs. Nevertheless, the preparation of SERS substrates encounters challenges owing to intricate and costly manufacturing procedures, rendering large-scale promotion unfeasible. To tackle this problem, Annasamy et al. devised a simpler and more cost-efficient SERS substrate. A SERS substrate was fabricated through the sequential deposition of graphene oxide–gold nanocomposites (GOAu), 2-mercaptoethanol, and glutaraldehyde onto a glass substrate, followed by antibody coating to yield the GOAu immunosubstrate. Upon the binding of 25(OH)D_3_ to the antibody, characteristic Raman peaks at 1223, 1253, and 1527 cm^−1^ were detectable under 785 nm excitation light. In comparison with the bare substrate, the SERS intensity of this GOAu immunosubstrate was enhanced by approximately fivefold. When integrated with portable devices and deep learning, this GOAu immunosubstrate was expected to expedite the clinical automated detection of 25(OH)D_3_ [114].

### 5.2. Smartphone-Assisted Imaging Platform

Smartphone-assisted imaging offers an innovative approach for the surveillance and quantitative assessment of substances, and it is anticipated to evolve into the next-generation portable diagnostic platforms.

Lee et al. devised an immunoassay apparatus termed vitaAID, which enables the rapid quantitative detection of 25(OH)D_3_ through the utilization of a smartphone. The vitaAID system encompasses iPhone accessories, a mobile application, and silver-enhanced LFSs. An iPhone was employed to capture the image data from both the test zone and the reference zone on the LFSs. The test zone was scanned to recognize the silver enhancement area, and a 100-pixel × 100-pixel area was selected around it. Subsequently, the average luminance of all pixels within this area was calculated. The identical procedure was replicated for the reference zone to ascertain its average luminance. By analyzing the luminance disparity between the test and reference zones and implementing the corresponding algorithm, the concentration of 25(OH)D_3_ was calculated [116]. Vemulapati et al. devised a TIDBIT for the purpose of capturing images on LFS and conducting an analysis of the T/C ratio (intensity of the test line to that of the control line). Utilized through a smartphone or computer in conjunction with the Nutriphone application, a Python script was employed to crop images and compute the T/C ratio. The T/C ratio was correlated with 25(OH)D_3_ by means of a pre-established calibration curve derived from samples with known characteristics. Through the integration of smartphone imaging technology and an alternative elution buffer, detection could be accomplished within 15 min, utilizing merely 40 μL of fingertip blood. Nevertheless, there remained a necessity for further enhancement in aspects such as the variability of image acquisition, the constraints of annotated datasets, and the achievement of accurate and precise quantitative analysis [112]. Anandita et al. devised a comprehensive algorithm for LFS diagnosis by utilizing ResNet-50 convolutional neural networks. This algorithm had the capacity to automatically scan, interpret, and quantify test images. By utilizing the smartphone camera, the results could be objectively documented and transmitted through a network connection for real-time reporting. When tested on commercial kits using images from controlled and smartphone environments, ResNet-50 demonstrated the highest accuracy (76.2%), a mean squared error of 0.00291, and a coefficient of determination (R^2^) of 0.9398, outperforming other models in terms of robustness. It was implemented for the quantification of 25(OH)D_3_ based on smartphones, thus laying the foundation for portable diagnostic applications in low-resource settings [117].

Smartphone-based biosensor analysis is not only applicable to LFSs but also stands as a State-of-the-Art innovation in the realm of electrochemical analyte detection. Sharma et al. devised a GO-modified electrode that can be inserted into a mobile phone, which was coupled with an Android system application to realize the quantitative detection of 25(OH)D_3_. GO was coated on the surface of a screen-printed paper electrode (SPPE), and subsequently, antibodies were modified on the electrode. CV and DPV were employed to detect the changes in the response current. Through integration with mobile devices and utilization of the application for real-time data analysis, the electrochemical analysis process was notably simplified. Owing to the integration of intelligent devices, this electrochemical platform facilitated on-site detection of 25(OH)D_3_ with a detection limit of 15 nM, offering a convenient and efficient approach for 25(OH)D_3_ detection [118].

### 5.3. Flexible Materials for Wearable Devices

Wearable sensors play a crucial role in personalized medicine, facilitating continuous data acquisition for the monitoring of health alterations to implement preventive interventions. Flexible electrode materials belong to a class of functional materials distinguished by remarkable mechanical flexibility and the ability to stably transmit electrical signals or support electrochemical reactions. They constitute the core basis for the development of electronic devices in the direction of “lightweight, flexible, and portable” designs and have been the focus of extensive and continuous research, particularly in the realm of wearable sensors. Screen printing enables the large-scale production of flexible electrodes on diverse substrates, rendering it highly suitable for applications in the wearable technology field. Martins et al. fabricated screen-printed carbon electrodes (SPCEs) on a flexible polyethylene terephthalate (PET) substrate. The SPCEs consisted of two carbon working electrodes and auxiliary electrodes, along with an Ag/AgCl electrode as a pseudoreference. L-cysteine was selectively adsorbed on gold planes to generate tree-like gold dendrites, which were then electrodeposited onto the SPCEs. AuDdrites demonstrate high electrical conductivity and biocompatibility, offering a large surface area for antibody immobilization, thereby facilitating the identification and capture of 25(OH)D_3_. The current during the detection process stems from the electro-oxidation of the redox probe ([Fe (CN)_6_]^3–/4–^). Upon the binding of the antibody to 25(OH)D_3_, the resultant immune complex functions as an insulating layer, partially impeding the diffusion of the redox probe, consequently leading to a current alteration [60]. SPCEs could be directly incorporated into immunosensor chips. By affixing the chip onto a mouthguard, it could serve as a wearable sensor. To further optimize flexible electrodes, encompassing enhancing precision, improving efficacy, and reducing expenses, Martins et al. reintroduced a disposable, label-free, or redox probe-free flexible bioelectronic chip based on PET. This chip integrated graphitic carbon nitride (CN) with Black Pearls 2000 carbon for the purpose of monitoring the level of 25(OH)D_3_ (Figure 6B). The chip was fabricated on a flexible polyethylene terephthalate (PET) substrate and comprised a working electrode, an auxiliary electrode, and pseudoreference electrodes (C_pse_). The working electrode was prepared using graphitic CN modified with toluidine blue (TB) and adorned with electrodeposited gold nanoparticles (Au-TB/CN), and was coated with a layer of anti-25(OH)D_3_ antibodies. Following gold electrodeposition, the C_pse_ was modified using Ag/AgCl conductive ink. 25(OH)D_3_ bound to antibodies to form a complex, which adhered to the electrode and creates an insulating layer, thus impeding electron transfer on the surface of the Au-TB/CN nanocomposite. By means of chronoamperometry, 25(OH)D_3_ could be detected at concentrations ranging from 0.25 to 1747 nM, with a detection limit of 0.025 nM. The low-cost characteristic significantly enhanced the appeal of this new flexible electrode as a real-time monitoring wearable device [115]. Beyond these PET-based flexible chips, carbon cloth (CC) could also be employed in wearable devices to realize real-time monitoring. CC, a flexible textile material, facilitates electrolyte diffusion. It was distinguished by high conductivity, chemical stability, light weight, low cost, and a three-dimensional structure. CC was mainly utilized in supercapacitor applications. Chauhan et al. fabricated a cerium nitrate (nCeO_2_)-anchored carbon cloth (CC) electrode for the electrochemical, label-free detection of 25(OH)D_3_. The nCeO_2_ was anchored onto the carbon cloth substrate to acquire a nanoelectrode (nCeO_2_/CC). Subsequently, antibodies and bovine serum albumin were immobilized on the nCeO_2_/CC for the recognition and capture of 25(OH)D_3_. The complex formed through the binding of the antibodies to 25(OH)D_3_ generated an electrically insulating layer on the electrode surface, impeding electron transfer. This modified CC-based nanobioplatform exhibited a detection limit of 11.6 nM and a response time of 15 min, conferring advantages on the fabrication of wearable sensors [119]. Flexible substrates and probe-free characteristic chips exhibited substantial potential in the advancement of wearable sensors. These investigations would stimulate researchers to devise wearable electrochemical immunoassay sensors with an extended detection scope, offering a prospective approach for the continuous surveillance of in vivo biological or chemical constituents. Nevertheless, augmenting the anti-interference ability in complex matrices persists as a challenge.

## 6. Conclusions and Outlook

The deficiency of vitamin D has emerged as a global epidemic, and the demand for monitoring its levels within the human body has been on the rise. As a pivotal biomarker for evaluating vitamin D levels in humans, the accurate detection of 25(OH)D_3_ is of utmost significance for the early diagnosis of metabolic bone diseases, including rickets and osteoporosis, as well as for promoting research in immune regulation and cardiovascular health. Although the existing literature has discussed the progress of detection methods for 25(OH)D_3_, there is a certain dearth of summarization and comparison of these methods, along with exploration of their practical application scenarios. This paper focuses on a systematic review of the latest advancements in 25(OH)D_3_ detection technologies based on antibody, aptamer, and molecularly imprinted recognition technologies. It identifies the areas requiring improvement in different detection and analysis methods and elaborates on the advantages and limitations of the existing techniques. Traditional detection technologies necessitate skilled personnel, specialized environments, and advanced equipment, which may be inaccessible in resource-constrained or emergency situations. In contrast, biosensors present multiple advantages in terms of sensitivity, selectivity, stability, and cost, and hold the potential for miniaturization and integration into portable devices. Nevertheless, further research is still required to optimize these biosensors to enhance their detection performance and practicality.

Biosensor-based detection methods for 25(OH)D_3_ possess substantial scientific significance and practical applicability in medical diagnostics and health management. Nevertheless, the detection process generally depends on blood samples. For test-subjects, blood collection is invasive and frequently painful, especially for infants, the elderly, and those who undergo frequent tests, thereby augmenting their susceptibility to infection. Moreover, the stability of blood samples is another crucial factor. If not processed in a timely manner, alterations in sample composition can influence its stability, resulting in compromised test outcomes and misjudgment of vitamin D levels, which can have a considerable impact on diagnosis and treatment. Consequently, the advancement of non-invasive sampling technologies has emerged as an urgent imperative.

In comparison to antibody-based biosensors, aptamer-based biosensors surmount the constraints of antibody instability and stringent storage prerequisites through the utilization of high-affinity nucleic acid aptamers for specific binding to target molecules. These biosensors significantly curtail detection expenses and operational intricacy. Their miniaturized configuration facilitates the analysis of microsamples (e.g., fingertip blood and saliva), augmenting the convenience of testing, particularly for primary care environments and at-home self-testing. However, in terms of sensitivity, both antibody-based and aptamer-based recognition, when combined with electrochemical techniques, can achieve picomolar-level detection. In contrast, MIP_S_ recognition can only reach micromolar-level detection. Overall, although biosensors have demonstrated good performance, there is still room for improvement in detecting low-concentration samples. To further elevate sensitivity, nucleic acid amplification techniques can be integrated with aptamer sensors. These amplification technologies can amplify the nucleic acid signals associated with the aptamer, thereby heightening the detection sensitivity for low levels of 25(OH)D_3_. This advancement would fulfill the clinical requirement for high-sensitivity detection in early diagnosis and disease prevention, thus paving new paths for the development of 25(OH)D_3_ detection technologies.

Portable and intelligent detection technologies relying on smartphones and wearable devices are anticipated to become a key area of future research. These devices not only enable real-time and non-invasive monitoring of 25(OH)D_3_ but also promote integration with cloud-based medical systems via the Internet of Things, thereby facilitating the establishment of personalized health management ecosystems. For instance, the combination of wearable biosensors with smartwatches is expected to enable continuous surveillance of vitamin D levels in the body and offer personalized supplementation suggestions. Micro-detection modules incorporated into smartphones, connected to cloud servers, can automatically upload home self-test data to medical platforms for remote diagnosis by medical professionals. Additionally, we must consider that vitamin D deficiency in older adults may lead to atherosclerosis [120], and cardiovascular and metabolic disorders [121]. The design of intelligent devices tailored for older adults, who might encounter operational challenges, necessitates a balance between user-friendly simplicity and intelligent functionality.

Looking forward, the standardization and harmonization of 25(OH)D_3_ detection methods ought to be continuously enhanced to augment the comparability of results among diverse techniques. This is expected to offer unified data support for global research on vitamin D in relation to health and disease prevention. Ranging from laboratory-based investigations to home health surveillance, and from clinical diagnoses to public health governance, the advancements in 25(OH)D_3_ detection technologies are anticipated to empower the broader health domain, driving precision medicine and personalized health management to new levels.

## Data Availability

No new data were created or analyzed in this study. Data sharing is not applicable to this article.

## Abbreviations

The following abbreviations are used in this manuscript:

1,25(OH)_2_D_3_	1,25-dihydroxyvitamin D_3_
25(OH)D_3_	25-hydroxyvitamin D_3_
3D	three-dimensional
Ab	monoclonal antibody
AgCNT/GCE	Ag-Ag_2_O/CNT-modified glassy carbon electrode
AgNV	Ag nanovilli
APCI	atmospheric-pressure chemical ionization
Asp	aspartic acid
AuNPs	gold nanoparticles
AuNRs	gold nanorods
BLI	Biolayer Interferometry
CAEF	electrospun cellulose acetate fiber
CC	carbon cloth
CdS	cadmium sulfide
CHA	catalytic hairpin assembly
CLIA	chemiluminescent immunoassay
CN	carbon nitride
COVID-19	coronavirus disease 2019
C_pse_	pseudoreference electrode
CRISPR	clustered regularly interspaced short palindromic repeats
CYP27	cytochrome P450 27
CYP450	cytochrome P450
DBP	D-binding protein
DCF	double-clad fiber
DPV	differential pulse voltammetry
dsDNA	double-stranded DNA
ECL	electrochemiluminescence
ELISA	enzyme-linked immunosorbent assay
EIS	electrochemical impedance spectroscopy
Fc-CHO	ferrocene-carbaldehyde
FTIR	Fourier-transform infrared spectroscopy
FRET	Förster resonance energy transfer
GCEs	glassy carbon electrodes
GdNPs	gadolinium nanoparticles
Gd_2_O_3_NRs	gadolinium oxide nanorods
GNR	graphene nanoribbon
GO	graphene oxide
GOAu	graphene oxide–gold nanocomposites
GQDs	graphene quantum dots
HCC	HOFs-g-C_3_N_4_-CeO_2_
HPLC	high-performance liquid chromatography
IDS-RIA	immunodiagnostic systems radioimmunoassay
ITO	indium tin oxide
LBD	ligand-binding domain
LC-MS	liquid chromatography–mass spectrometry
LC-MS/MS	liquid chromatography–tandem mass spectrometry
LED	light-emitting diode
LFS	lateral flow strip
LLE	liquid–liquid extraction
LOD	limit of detection
LSPR	localized surface plasmon resonance
MB	methylene blue
ME HLB	membrane-emulsified hydrophilic–lipophilic balance
MIPs	molecularly imprinted polymers
MNBP	microfluidic nanobioplatform
MPNs	mesoporous platinum nanozymes
MS	mass spectrometry
N-CNT	CuCo_2_O_4_/nitrogen-doped carbon nanotube
OF	optical fiber
Pd-Co NPs	palladium-doped cobalt nanoparticles
PDMS	polydimethylsiloxane
PET	polyethylene terephthalate
PG	PicoGreen
POCT	point-of-care testing
PPT	protein precipitation
PPy	polypyrrole
PTAD	4-phenyl-1,2,4-triazoline-3,5-dione
QDs-FICA	dot nanoparticle-based fluorescent immunochromatographic assay
RIA	radioimmunoassay
RCP	conducting paper substrate
Rct	charge transfer resistance
SELEX	systematic evolution of exponentially enriched ligands
SERS	surface-enhanced Raman scattering
SLDB	split-luciferase vitamin D biosensor
SPCEs	screen-printed carbon electrodes
SPE	solid-phase extraction
SPPE	screen-printed paper electrode
SPR	surface plasmon resonance
SWV	square wave voltammetry
TMB	3,3′,5,5′-tetramethylbenzidine
TSA	trident scaffold-assisted aptamer biosensor
TB	toluidine blue
UV	ultraviolet
UV-vis	ultraviolet–visible spectroscopy
VD	vitamin D
VD_2_	vitamin D_2_
VD_3_	vitamin D_3_
VDR	vitamin D receptor
